# Physiological, Photosynthetic, and Transcriptomics Insights into the Influence of Shading on Leafy Sweet Potato

**DOI:** 10.3390/genes14122112

**Published:** 2023-11-22

**Authors:** Xiaojing Jing, Peiru Chen, Xiaojie Jin, Jian Lei, Lianjun Wang, Shasha Chai, Xinsun Yang

**Affiliations:** 1 Institute of Food Crops, Hubei Academy of Agricultural Sciences, Wuhan 430064, China; 2021710708@yangtzeu.edu.cn (X.J.); 15592209837@163.com (P.C.); xiaojiejin@hbaas.com (X.J.); leijian2006@hbaas.com (J.L.); wanglianjun@hbaas.com (L.W.); chaishasha2008@hbaas.com (S.C.); 2 Agricultural College, Yangtze University, Jingzhou 434022, China

**Keywords:** leafy sweet potato, leaf growth, transcriptome, shading treatment

## Abstract

Leafy sweet potato is a new type of sweet potato, whose leaves and stems are used as green vegetables. However, sweet potato tips can be affected by pre-harvest factors, especially the intensity of light. At present, intercropping, greenhouse planting, and photovoltaic agriculture have become common planting modes for sweet potato. Likewise, they can also cause insufficient light conditions or even low light stress. This research aimed to evaluate the influence of four different shading levels (no shading, 30%, 50%, and 70% shading degree) on the growth profile of sweet potato leaves. The net photosynthetic rate, chlorophyll pigments, carbohydrates, and polyphenol components were determined. Our findings displayed that shading reduced the content of the soluble sugar, starch, and sucrose of leaves, as well as the yield and Pn. The concentrations of Chl a, Chl b, and total Chl were increased and the Chl a/b ratio was decreased for the more efficient interception and absorption of light under shading conditions. In addition, 30% and 50% shading increased the total phenolic, total flavonoids, and chlorogenic acid. Transcriptome analysis indicated that genes related to the antioxidant, secondary metabolism of phenols and flavonoids, photosynthesis, and MAPK signaling pathway were altered in response to shading stresses. We concluded that 30% shading induced a high expression of antioxidant genes, while genes related to the secondary metabolism of phenols and flavonoids were upregulated by 50% shading. And the MAPK signaling pathway was modulated under 70% shading, and most stress-related genes were downregulated. Moreover, the genes involved in photosynthesis, such as chloroplast development, introns splicing, and Chlorophyll synthesis, were upregulated as shading levels increased. This research provides a new theoretical basis for understanding the tolerance and adaptation mechanism of leafy sweet potato in low light environments.

## 1. Introduction

Sweet potato (*Ipomoea batatas* [L.] Lam) is the sixth most important food crop in the world and it is widely cultivated in over 100 countries, especially China, which is the largest producer [1,2]. Apart from its conventional storage roots, the young leaf tips of sweet potato (initial 10–15 cm from the growing end) can also be eaten as a green vegetable [3,4]. Researchers have reported that sweet potato greens are good sources of proteins, dietary fibers, minerals, and vitamins [5,6,7]. Furthermore, sweet potato greens have high contents of phenolic compounds, especially caffeoylquinic acid derivatives, such as chlorogenic acid, 3,4-di-O-caffeoylquinic acid, 3,5-di-O-caffeoylquinic acid, 4,5-di-O-caffeoylquinic acid, and 3,4,5-tri-O-caffeoylquinic acid [4,8,9]. And these phenolics can provide antioxidant properties and mean that leafy sweet potato has various health benefits such as anti-aging, anti-cancer, anti-bacterial, anti-inflammatory, anti-hypertensive, and anti-diabetic (type II) effects [10,11,12,13]. In addition, leaf sweet potato has high adaptability and could be harvested several times a year due to its short production cycle [14].

Due to limited land resources, intercropping becomes a common cultivation pattern for sweet potato. In China, sweet potato is often intercropped with corn, tobacco, sesame, fruit trees, etc. [15,16,17]. Recently, sweet potato is used in photovoltaic agriculture, and planted under photovoltaic panels [18]. However, sweet potato is a photophile crop and may be subjected to insufficient solar radiation or even low light stress when planted under these planting modes [19]. In addition to being the primary energy source for photoautotrophic higher plants, light also controls multiple developmental processes throughout the plant life cycle, including phototropism, shade avoidance, circadian rhythms, and flowering time, etc. [20]. Thus, these cultivation patterns affect sweet potato growth, yield, and quality traits due to shading conditions. He et al. [19] reported that shading reduced storage root yield but increased the anthocyanin output of purple-flesh sweet potato. Park et al. [21] evaluated the growth and yield of two sweet potato cultivars under shading conditions, and the results showed that both cultivars tended to suppress tuber root growth, and caused yield reduction. Wei et al. [22] reported that photovoltaic panel shading decreased the yield per unit area of three sweet potato cultivars (Xinxiang, Zheshu 13, and Zheshu 77) by 39.25%, 33.70%, and 23.60%, respectively. Lately, a few studies were published on plant responses to shading in terms of genes and transcriptional expression levels [23,24,25,26]. Zhang et al. [23] revealed that shading had an impact on porphyrin and chlorophyll metabolism, and on mined genes related to pigment synthesis in elm. Ma et al. [24] observed that shading reduced the variety and concentration of volatile organic compounds (VOCs) by affecting fatty acid, amino acid, and the isoprene pathways in grape berry. Zaman et al. [26] reported that the genes expression linked with photosynthesis and the MAPK pathway were enhanced by shading and mediated the cold tolerance of tea. However, the growth characteristics, physiological response, and related gene expression changes in leafy sweet potato in response to shading conditions are still unclear.

To better understand the effects of shading on leafy sweet potato, a leafy sweet potato cultivar Eshu 10 was grown under four differential shading conditions (0%, 30%, 50%, and 70% shading degree), and its physiological and photosynthetic characteristics were determined. Meanwhile, differentially expressed genes (DEG) between shading and CK (full sunlight) were screened via transcriptome analysis.

## 2. Materials and Methods

### 2.1. Plant Materials and Growth Conditions

The leafy sweet potato cultivar Eshu 10 (GPD Sweet Potato (2018) 420052) was used in this experiment, and grown in an experimental field in the Hubei Academy of Agricultural Sciences (N 30°29′, E 114°18′) in Wuhan, Hubei, China in 2021, with a subtropical humid monsoon climate. Eshu 10 was widely cultivated in Hubei province with high-yield and tender taste characteristics. Seedlings of 15–20 cm cut with scissors were transplanted into the experimental field on 21 May; the soil type was yellow-brown soil. Field management was carried out based on the standard agricultural practices. 

Twenty days after planting (DAP), the plants passed the slow seedling stage and entered the growth stage, and were subjected to three levels of natural irradiance by using plastic nets with different light transmittance. The plants with full sunlight (0% shade) were used as a control, the light intensity of each treatment was measured using a Lighter meter (LX-1128SD, Lutron), and the shading rates were 30%, 50%, and 70% (representing light, moderate, and severe shade, respectively) [21]. Each treatment was replicated three times and each replicate contained 32 plants. At 65 DAP, the tips (10–15 cm below shoot tip growth point) were collected with scissors, and the top four fully expanded leaves of leafy sweet potato were used for measuring the physiological and photosynthetic parameters. Meanwhile, the samples were collected and immediately frozen in liquid nitrogen, and stored at −80 °C for further use. Each sample has three biological replicates, and each biological replicate contains 8 plants.

### 2.2. Measurement of Photosynthesis

The net photosynthetic rate (Pn) (μmol m^−2^ s^−1^) was measured by using a portable photosynthesis system (LI-6400, LI-COR, Lincoln, NE, USA) from 9:00 to 11:00. The instrument parameters were set as follows: a steady light intensity of 1200 μmol m^−2^ s^−1^, CO_2_ concentration of 380 μmol mol^−1^.

### 2.3. Measurement of Chlorophyll Contents

Leaf discs (0.2 g) from the top four fully expanded leaves were dipped in 20 mL 95% ethanol in the dark for 48 h at room temperature. Thereafter, the supernatant was measured using a spectrophotometer at wavelengths of 665 and 649 nm to evaluate the chlorophyll a and b concentrations.

### 2.4. Measurement of Soluble Sugar, Sucrose, and Starch Contents

Dried leaf powder (0.1 g) was used to homogenate with 5 mL 80% ethanol, and then placed in a water bath for 20 min at 80 °C. After cooling to room temperature, the homogenate was centrifuged at 4000 rpm for 5 min. The supernatant was transferred to a 50 mL volumetric flask. We repeated the above steps twice, and the supernatants were collected together and we fixed the volume to 50 mL (solution A). After that, 4 mL ddH2O and 2 mL 9.2 M perchloric acid were added the residue, and kept in a water bath for 20 min. After cooling, the samples were centrifuged at 4000 rpm for 10 min, and the supernatant was collected in a 50 mL volumetric flask. The residue was washed with ddH2O twice, and the supernatants were collected and we fixed the volume to 50 mL (solution B). Finally, solution A was used to determine soluble sugar and sucrose content, while Solution B was used to determine starch content. The absorbance was measured at 620 nm using a UV-2880 spectrophotometer (UNICO, Shanghai, China). The calibration curve of glucose and sucrose (ranging from 0 to 0.1 mg/mL) was prepared; ddH2O was used as control.

### 2.5. Total Phenolics and Flavonoids Determination

Total phenolics were extracted using 1.0 g dried leave powder, 70% ethanol, and ultrasonic wave methods, as described by Sun et al. [27]. The total phenolic content (TPC) was determined according to the Folin–Ciocalteu method [28]. A total of 1 ml prepared solution was placed in a 10 mL tube, 1 mL Folin–Ciocalteu reagent (Guoyao, Shanghai, China), 3 mL 7.5% Na_2_CO_3_ and 5 ml ddH2O were added, and then placed in a water bath for 1.5 h at 45 °C. The absorbance was measured at 765 nm using a UV-2880 spectrophotometer (UNICO, Shanghai, China). A calibration curve of gallic acid (ranging from 0 to 0.05 mg/mL) (Guoyao, Shanghai, China) was prepared; TPC was expressed as mg gallic acid equivalent on a dry weight basis (mg GAE/g DW).

Total flavonoids were extracted using 1.0 g dried leave powder, 60% ethanol, and ultrasonic methods. The total flavonoid content (TFC) was assayed using the colorimetric aluminum chloride method [29], 1 mL prepared solution was placed in a 10 mL tube, 1 mL 60% ethanol, 0.5 mL 5% NaNO_2_, 0.5 mL 10% Al(NO_3_)_3_, 4 mL 4% NaOH, and 4 mL ddH2O were added, with a 15 min static reaction time. The absorbance was measured at 510 nm using a UV-2880 spectrophotometer (UNICO, Shanghai, China). A calibration curve of rutin (ranging from 0 to 0.04 mg/mL) (Guoyao, Shanghai, China) was prepared; TFC was expressed as mg rutin equivalent on a dry weight basis (mg RE/g DW).

### 2.6. Chlorogenic Acid Determination

Chlorogenic acid was extracted from the leaves as described in a previous study [30]. A total of 1 mL extract was placed in a 10 mL tube, and 9 mL 95% ethanol was added. The sample was then measured at 333 nm using a UV-2880 spectrophotometer (UNICO, Shanghai, China). The chlorogenic acid concentration was calculated using a standard solution of commercial chlorogenic acid. A calibration curve of chlorogenic acid (ranging from 0 to 15 μg/mL) (Yuanye Bio-Technology Co., Shanghai, China, HPLC ≥ 98%) was prepared. 

### 2.7. Transcriptome Analysis

Total RNA of 4 samples (CK, L30, L50, L70 represents 0%, 30%, 50%, 70% shading degree) was isolated using TRIzol reagent (Invitrogen, Carlsbad, CA, USA), and sent to Shanghai Major Bio-pharm Biotechnology Co., Ltd. (Shanghai, China) for transcriptome library construction and sequenced on Illumina NovaSeq 6000 platform.

After quality control, clean reads were mapped to the reference genome “Taizhong 6” (https://121.36.193.159/download_genome.html, accessed on 28 January 2022) using HISAT2 (v2.1.0) software [31]. Gene expressions were calculated according to fragments per kilobase per million mapped reads (FPKM). DEG were screened using DESeq2 [32] with the criteria of |log2FC (fold change)| ≥ 1 and *p*-value < 0.01.

### 2.8. qRT-PCR

Primers of four DEGs were designed using Primer 5, and β-actin was used as the reference gene (Supplementary Table S1). qRT-PCR was performed on 7500 Real-Time PCR system (Applied Biosystems, Foster City, CA, USA). The relative expression levels of genes were calculated using the 2^−ΔΔCt^ method [33]. For each sample, three biological replicates and three technical replicates were performed.

### 2.9. Statistical Analysis

Variance analysis of physiological and photosynthetic traits under different shading conditions was performed using Student’s *t*-test. The threshold of significant difference was set to *p*-value < 0.05.

## 3. Results

### 3.1. Effect of Shading on Yield of Leafy Sweet Potato

The yield of leafy sweet potato cultivar Eshu 10 under different shading conditions is shown in Table 1. There was a significant reduction in the yield at all shading levels, the rank-order being control > 30% shading > 50% shading > 70% shading. This indicated that the crop yield of Eshu 10 was significantly affected by shading stress.

### 3.2. Effect of Shading on Chlorophyll Concentration and Pn of Leafy Sweet Potato

**Figure 1:**
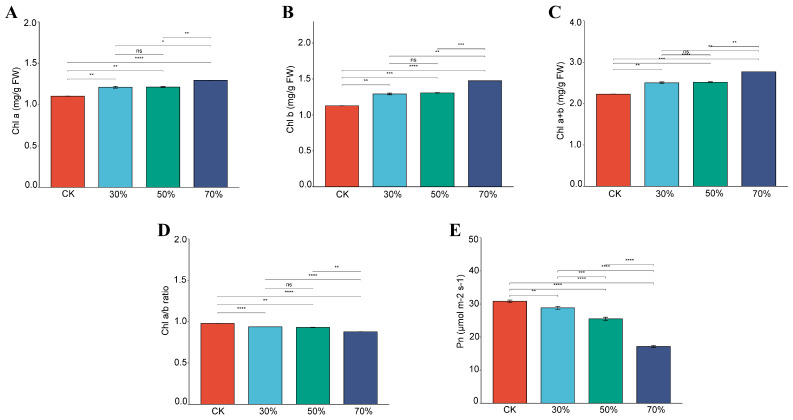
Effect of shading on chlorophyll concentration and Pn of the four fully expanded leaves of leafy sweet potato. (**A**): Chl a concentration; (**B**): Chl b concentration; (**C**): total Chl concentration; (**D**): Chl a/b ratio; (**E**): Pn. CK: full sunlight; 30%: 30% shading; 50%: 50% shading; 70: 70% shading. *, **, ***, and **** significant at the 0.05, 0.01, 0.001, and 0.0001 probability levels, respectively; ns: no significant difference.

Compared with full sunlight, sweet potato leaves grown under shading conditions had significantly higher concentrations of Chl a, Chl b, and total Chl. The leaves under 70% shading had the highest Chl concentrations, followed by 50%, and 30% shading (Figure 1). However, the leaves under full sunlight had the highest Chl a/b ratio, followed by those grown under 30%, 50%, and 70% shading degrees. Interestingly, there were no significant differences in the above chlorophyll traits between 30% and 50% shading.

The net photosynthetic rate (Pn) of leafy sweet potato cultivar Eshu 10 under different shading conditions is shown in Figure 1E. The Pn was significantly decreased with the increase in shading degree. Plants under full sunlight had the highest Pn, while those grown under a 70% shading degree had the lowest Pn. This indicated that the Pn of Eshu 10 was significantly affected by shading stress.

### 3.3. Effect of Shading on Soluble Sugar, Sucrose, and Starch of Leafy Sweet Potato

**Figure 2:**
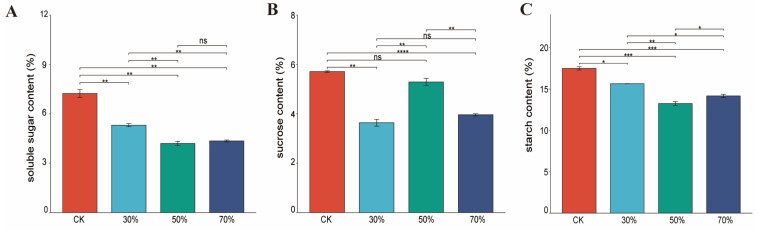
Effect of shading on carbohydrate contents of the four fully expanded leaves of leafy sweet potato. (**A**): soluble sugar; (**B**): sucrose; (**C**): starch. *, **, ***, and **** significant at the 0.05, 0.01, 0.001, and 0.0001 probability levels, respectively; ns: no significant difference.

The contents of soluble sugar and starch decreased significantly as the shade treatment degree increased. More specifically, the rank-order for soluble sugars and starch contents was control > 30% shading > 70% shading > 50% shading, the soluble sugars and starch contents were prompted by 3.57% and 6.95% at 70% shading over 50% shading (Figure 2). Shading also decreased the sucrose contents and showed an interesting trend in response to shading treatments, the rank-order for sucrose contents was control > 50% shading > 70% shading > 30% shading, and the sucrose content under 50% shading was enhanced by 59.82% and 33.59% over 30% and 70% shading, respectively. Hence, shade conditions were detrimental to the accumulation of carbohydrates.

### 3.4. Effect of Shading on TPC, TFC, and Chlorogenic Acid in Leafy Sweet Potato

**Figure 3:**
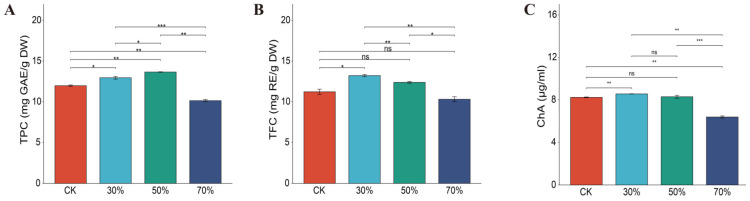
Effect of shading on phenolic compounds contents of the four fully expanded leaves of leafy sweet potato. (**A**): total phenolic content; (**B**): total flavonoid content; (**C**): chlorogenic acid content. *, **, and *** significant at the 0.05, 0.01, and 0.001 probability levels, respectively; ns: no significant difference.

To explore the effect of shading stress on the polyphenol components of Eshu 10, we determined the contents of total phenols, chlorogenic acid, and total flavonoids in leaves, respectively. As shown in Figure 3, the TPC content was significantly changed under different shading conditions with the rank-order of 50% shading > 30% shading > control > 70% shading, whose content for 50% shading was 1.14-times, 1.05-times, and 1.35-times greater than that of the control, 30%, and 70% shading conditions, respectively. Compared with CK, there were no significant changes in TFC under 50% and 70% shading, and the TFC content for 30% shading was 1.18-times, 1.07-times, and 1.28-times greater than that of control, 50%, and 70% shading conditions. Similar to TFC, the rank-order for ChA content was also 30% shading > 50% shading > control > 70% shading, 30% shading, and 70% shading could significantly increase or decrease ChA content, respectively. This indicated that 30% and 50% shading had a favorable effect on polyphenols, but 70% shading significantly decreased them as compared to CK.

### 3.5. Transcriptome Analysis of Leaves under Different Shading Treatments

RNA-Seq were carried out using leaves of the plants under four shading treatments. After removing the unknown reads, low-quality reads, and adaptor sequences, approximately 88.08 Gb of clean data were obtained from 12 libraries, with the Q30 percentages (high-quality sequences) ranging from 94.15 to 94.68% (Supplementary Table S2). The clean data were used for reads mapping, with the total reads mapping rate ranging from 83.22 to 85.65% (Supplementary Table S2), indicating that the transcriptome data met the requirements for further analysis.

**Figure 4:**
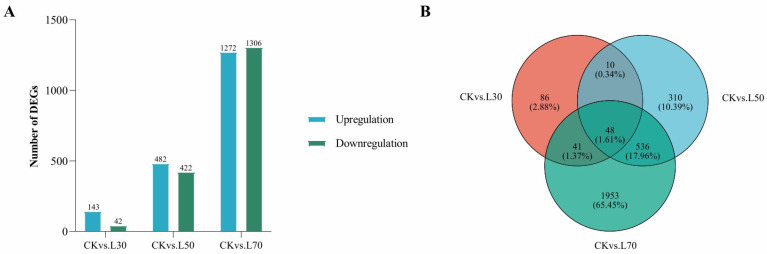
Identification of differentially expressed genes (DEGs) of Eshu 10 under shading treatments. (**A**): The number of DEGs by comparing CK vs. L30, CK vs. L50, CK vs. L70; (**B**): Venn diagrams of DEGs among the three comparisons. CK, L30, L50, and L70 represent the samples under different shading treatments (0%, 30%, 50%, and 70% shading degree).

**Figure 5:**
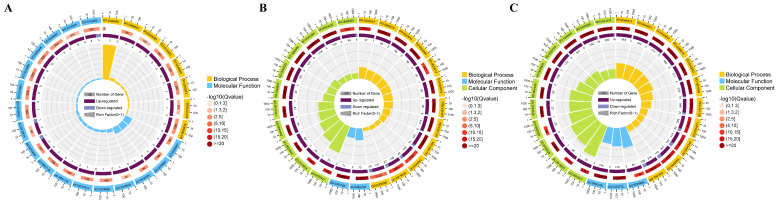
Results of GO enrichment analyses of DEGs from CK vs. L30, CK vs. L50, and CK vs. L70. (**A**): CK vs. L30; (**B**): CK vs. L50; (**C**): CK vs. L70. Top 25 enriched GO terms were displayed.

**Figure 6:**
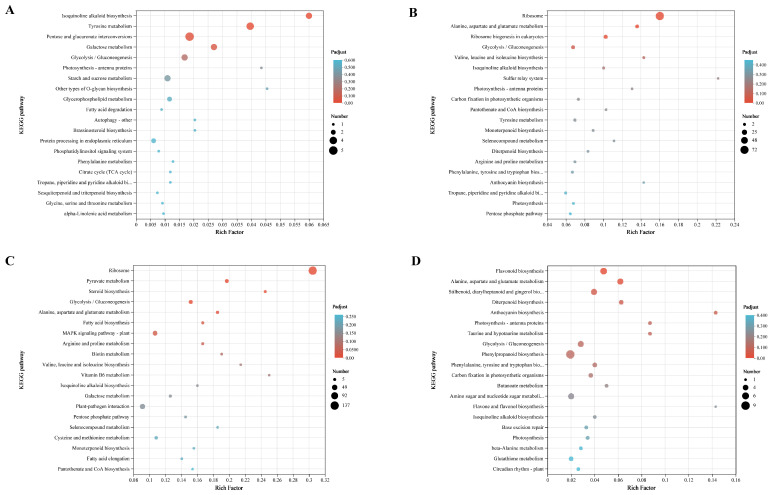
Results of KEGG enrichment analyses of CK vs. L30, CK vs. L50, CK vs. L70, and comparison-specific DEGs of CK vs. L50. (**A**): CK vs. L30; (**B**): CK vs. L50; (**C**): CKvs. L70; (**D**): Comparison-specific DEGs of CK vs. L50. The top 20 enriched pathways were displayed.

**Figure 7:**
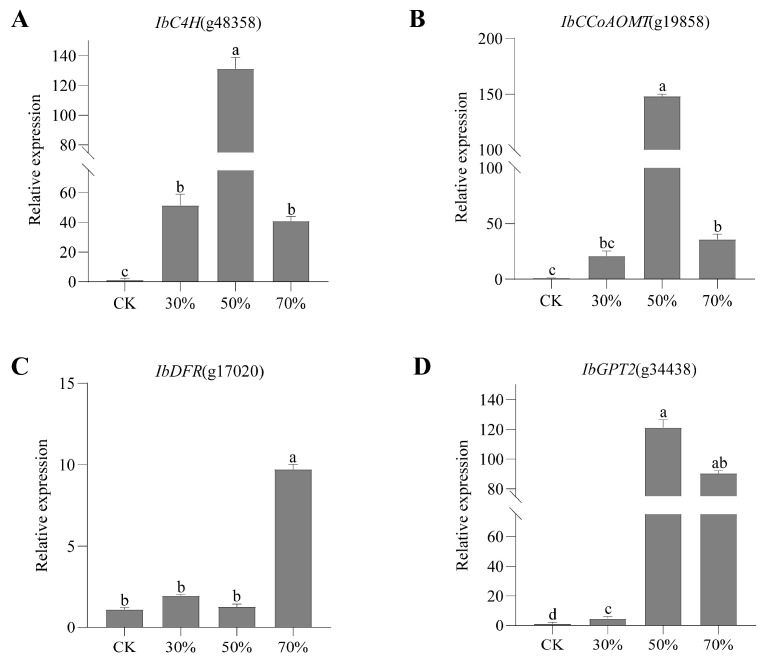
The validation of RNA-Seq data of 4 DEGs via qRT-PCR. (**A**–**D**):Expression patterns of IbC4H, IbCCoAOMT, IbDFR, and IbGPT2. Lowercase letters indicate a significant difference (*p* ≤ 0.05).

Transcriptomes at 30%, 50%, and 70% shading conditions were compared with those at CK conditions to identify DEGs. A total of 185, 904, and 2578 DEGs were identified in three comparisons of CK_vs_L30, CK_vs_L50, and CK_vs_L70, respectively (Supplementary Table S3; Figure 4). Among them, 48 DEGs were shared in three comparisons, while 86, 310, and 1953 DEGs were comparison-specific DEGs (Figure 4). The GO enrichment analyses classified the DEGs from three comparisons into cellular component (CC), molecular function (MF), and biological process (BP) (Figure 5). The DEGs of CK_vs_L30 were significantly enriched in antioxidation-related GO terms “oxidoreductase activity, acting on peroxide as acceptor (GO:0016684)”, “peroxidase activity (GO:0004601)”, and “glutathione peroxidase activity (GO:0004602)”. CK_vs_L50 and CK_vs_L70 contain many shared significant GO terms such as “structural constituent of ribosome (GO:0003735)” and “translation (GO:0006412)”. KEGG enrichment analyses suggested that 36, 103, and 113 KEGG pathways were enriched in CK_vs_L30, CK_vs_L50, and CK_vs_L70, respectively. “Isoquinoline alkaloid biosynthesis (map00950)” was only significantly enriched in CK_vs_L30, while “Ribosome (map03010)” was significantly enriched in CK_vs_L50 and CK_vs_L70 (Figure 6). Furthermore, “Plant hormone signal transduction (map04075)” and “Photosynthesis-antenna proteins (map00196)” could be identified in all comparisons. KEGG enrichment analyses were also conducted on the specific DEGs in the above-mentioned comparisons, showing that the comparison-specific DEGs of CK_vs_L50 were uniquely enriched in the secondary metabolism of phenol- and flavonoid-related pathways, including “Flavonoid biosynthesis (map00941)”, “Stilbenoid, diarylheptanoid and gingerol biosynthesis (map00945)”, “Anthocyanin biosynthesis (map00942)”, “Phenylpropanoid biosynthesis (map00940)”, “Phenylalanine, tyrosine and tryptophan biosynthesis (map00400)”, and “Flavone and flavonol biosynthesis (map00944)” (Figure 6). Four DEGs related to these pathways were randomly selected for validation, which showed that the transcriptome expression trends were consistent with the qRT-PCR results (Figure 7), indicating that reliable RNA-seq data were obtained from 12 samples.

**Figure 8:**
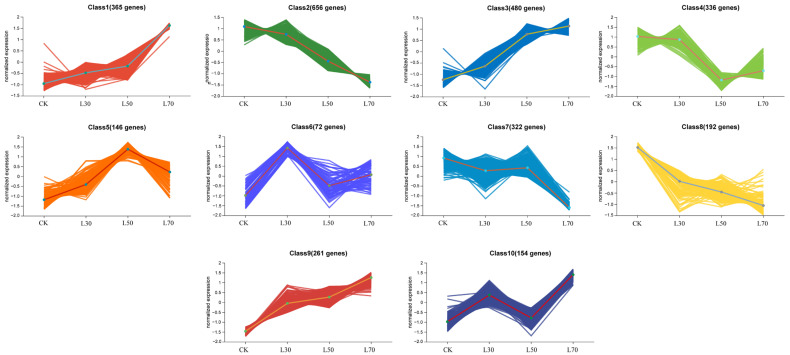
K-means cluster analysis of DEGs. The different colors lines represent the average pattern of all DEGs in each class.

To gain further insight into the gene-expression patterns under different shading conditions, all DEGs were divided into 10 clusters using the k-means clustering algorithm (Figure 8). In Eshu10, Class1 (365 genes), Class3 (480 genes), and Class9 (261 genes) showed an upward trend. Class2 (656 genes), Class4 (336 genes), Class7 (322 genes), and Class8 (192 genes) showed a downward trend. Class1, Class3, and Class9 were selected for further study due to genes in these clusters showing higher expression trends as the light intensity was reduced. GO enrichment analyses classified the DEGs into CC, BP, and MF, the results for three up-trend clusters exhibited significant differences in photosynthesis-related GO terms, such as “chloroplast (GO:0009507)”, “chloroplast stroma (GO:0009570)”, and “chloroplast envelope (GO:0009941)” (Supplementary Figure S1). According to KEGG enrichment analyses, 58, 74, and 46 pathways were enriched in Class1, Class3, and Class9, respectively. Most of the DEGs in these pathways, such as “Pyruvate metabolism (map00620)”, “Glycolysis/Gluconeogenesis (map00010)”, and “Ribosome (map03010)”, were significantly enriched (Supplementary Figure S2). Interestingly, “Porphyrin and chlorophyll metabolism (map00860)”, “Photosynthesis-antenna proteins (map00196)”, and “Photosynthesis (map00195)” were enriched in Class 9. And “Porphyrin and chlorophyll metabolism (map00860)” could be identified in all up-trend clusters. We focused on the DEGs that were significantly enriched in photosynthesis-related terms or pathways, which might play essential roles in coping with shading stress in Eshu 10. A total of 32 DEGs were collected, such as *DCL*, *RPL21c*, *DAG*, *SG1*, *FtsHi5*, *Tic110*, and *PPOX1*, etc. (Supplementary Table S4).

## 4. Discussion

In response to the intensification and sustainable development of agricultural production, greenhouse planting, intercropping, and photovoltaic agriculture have become common planting modes for sweet potato [14,17,18]. Likewise, they can also cause insufficient light conditions or even low light stress. So far, research on the impact of insufficient light on plants has mainly focused on the morphology, photosynthetic characteristics, and dry-matter accumulation, including various plant materials such as soybean, corn, tea tree, and tomato, with few reports in sweet potato [34,35,36]. In the present study, the leafy sweet potato cultivar Eshu 10 was selected as the experimental crop and we intended to study the effects of reduced light on the physiology, photosynthesis, and transcriptomics through artificial shading. 

### 4.1. Impact of Different Shading Treatments on Photosynthetic Characteristics and Carbohydrates Contents

Previous studies have shown that shading directly restricted the net photosynthetic rate of plants, due to the low interception of light in comparison to full sunlight conditions [37]. Chlorophyll a and b are important photosynthetic pigments of plants, due to their crucial roles in the absorption, transmission, and conversion of solar energy [38,39]. The present study revealed that shading stresses significantly decreased the net rate of photosynthesis but increased the contents of Chl a, Chl b, and total Chl of leaves of Eshu 10. And the higher shading degree, the higher Chl contents. In addition, the Chl a/b ratio was significantly decreased by shading density, suggesting richer Chl b had been produced in response to shading. Our results were like those of one previous study, which reported higher concentrations of total Chl and Chl b in shading groups than in a full sunlight group, and this phenomenon was stronger under severe shading than under moderate shading [40]. Another study revealed that, during the intercropping period, the photosynthesis activities of sweet potato were at a low level, and the total Chl, Chl a, and Chlb increased with the degree of shading, while the Chl a/b ratio decreased [41]. It was also demonstrated that Chl a was ubiquitous and directly involved in photosynthesis, while Chl b indirectly participates in photosynthesis by broadening the wavelength of absorbable light and capturing additional photons [42,43]. Moreover, a low ratio of Chl a+b encouraged plants to capture more photons over a wider range for photosynthesis supplement [40,43,44]. Therefore, under low light environments, the increase in Chl b and decrease in Chl a/b helped to improve the photosynthetic efficiency of leafy sweet potato. 

It has been testified that carbohydrates not only serve as quality components, but they also play pivotal roles as energy sources, structural components, and signaling molecules of plants under normal and stressed conditions [45]. Our results demonstrated that shade conditions were detrimental to the accumulation of carbohydrates, with the content of soluble sugar, starch, and sucrose of leaves of Eshu 10 decreasing irregularly. Our findings were similar to those previously reported in which shade stress diminished the sugar and starch metabolism levels of the leaves of sweet potato (Zhenghong 23); the contents of important soluble sugars such as sucrose, glucose, maltose, and raffinose also decreased to varying degrees, which led to a reduction in the carbon assimilates transported to the roots and to the obstruction of root expansion [46]. It was also elaborated that 20% shading promoted the synthesis of soluble sugars in purple-fleshed sweet potato leaves and root tubers, but the contents of soluble sugars decreased rapidly under 40–80% shading [47]. The yield of leafy sweet potato was composed of young leaf tips; the effect of shading on the root carbohydrates of Eshu 10 is not yet clearly understood.

### 4.2. Differentiation of Transcription Levels under Different Shading Conditions

When plants grow under abiotic stresses, such as salinity, flooding, drought, etc., reactive oxygen species (ROS) would accumulate in their bodies and they would suffer secondary damage [48]. Our findings indicated that antioxidant-related GO terms had a unique response to 30% shading stress; most of the upregulated DEGs were POD, PPO, GPX, and AAO family genes (Supplementary Table S5), which were responsible for eliminating ROS and maintaining the intracellular redox balance [49,50,51]. Our findings supported the previous study in which the total antioxidant activity and hydroxyl radical scavenging activity of sweet potato were significantly increased by light shading, but decreased rapidly under deeper shading conditions [47]. The above results indicated that 30% shading triggered the ROS scavenging system of Eshu 10. 

Flavonoids are one of the most important secondary metabolites participating in plant growth, development, and defense, and they have various therapeutic activities [52,53,54,55]. It had been proved that light shading promoted the synthesis of anthocyanins and flavonoids, which was consistent with our research results [47]. In the present study, 50% shading increased the total phenolic and total flavonoids, while 70% shading significantly reduced them. In addition, DEGs of CK_ Vs_ L50 were uniquely enriched in the secondary metabolism of phenol- and flavonoid-related pathways, and most of the DEGs were upregulated under 50% shading compared with CK, such as *C4H*, *CCoAOMT*, *CoMT*, *DFR*, *GPT2*, and *DHD-SHD* (Supplementary Table S6). Some other researchers have proved that the expression levels of some structural genes related to flavonoid synthesis could be altered by different light conditions, including *C4H*, *UFGT*, and *DFR* [56,57,58]. In addition, the first oxidation reaction to generate ρ-coumaric acid in flavonoids synthesis were catalyzed by *C4H* [55,59]. *CCoAOMT* and *DFR* contributed to the accumulation of flavonoids and enhanced plant stress resistance, while *GPT2* was necessary for photodynamic domestication [60,61,62]. These findings suggested that 50% shading induced a high expression of enzyme genes, which might increase the content of total flavonoids and total phenolic compounds.

The MAPK signal cascades played crucial roles in plant growth, and in plant immune and stresses response [63]. When exposed to 70% shading, the photosynthesis activities and physiological characteristics of Eshu 10 were strongly inhibited. On the other hand, the comparison-specific DEGs of CK_ Vs_ L70 were significantly enriched in the “MAPK signaling pathway (map04016)” (Supplementary Figure S3). Moreover, MAPK participated in the signal transduction pathways of various stress-related hormones such as ethylene, abscisic acid, and salicylic acid [64,65]. Our results showed that most of the DEGs were downregulated and enriched in the abscisic-acid-activated signaling pathway and ethylene signal transduction, such as *EIN3*, *ERF1*, *PYL2*, *PYL3*, *PYL4*, etc. In addition, some key defense response genes were also downregulated in our study, such as *BAK1*, *MAPKK2*, *SNRK2*, and *FLS2*. These results indicated that 70% shading caused extreme environmental stress.

### 4.3. Photosynthesis-Related DEGs in Eshu 10 Play an Important Role during Shading Conditions

Well-developed chloroplasts and the accumulation of chlorophylls are essential for photosynthesis [66,67]. The splicing process of introns affects the translation of chloroplast genes, and it is related to the assembly of photosystem complexes, thereby affecting photosynthesis [68,69]. The splicing factors include PPR proteins, CRM domain proteins, and the mTERF protein family [70,71,72,73]. Chlorophyll levels fluctuated significantly under low-light environments, as their biosynthesis required the participation of catalytic enzyme genes, such as *CHLI* and *PPOX*, which were highly expressed in the present study [74,75]. Other DEGs related to chloroplast development and Chlorophyll synthesis were also collected, such as *DCL*, *RPL21c*, *DAG*, *SG1*, *FtsHi5*, and *Tic110* [76,77,78,79,80,81]. Furthermore, the introns-splicing-related DEGs, such as PPRs, CFM3, MTERF4, and MTERF5, were also upregulated in this study. These DEGs involved in chloroplast development, Chlorophyll synthesis, and introns splicing might be indispensable for Eshu 10 to adapt to low-light conditions. 

## 5. Conclusions

This research verified that shading levels had significant effects on the physiological and photosynthetic properties of the leafy sweet potato cultivar Eshu 10. Specifically, shading significantly reduced the yield and Pn of Eshu 10, as well as the contents of soluble sugar, starch, and sucrose of leaves. The concentrations of Chl a, Chl b, and total Chl were increased and the Chl a/b ratio was decreased for a more efficient interception and absorption of light under shading. And 30% and 50% shading increased the total phenolic, total flavonoids, and chlorogenic acid. Transcriptome analysis indicated that the genes related to the antioxidant, secondary metabolism of phenols and flavonoids, photosynthesis, and the MAPK signaling pathway were altered in response to shading stresses. This work provides a new theoretical basis for understanding the tolerance and adaptation mechanism of leafy sweet potato in low-light environments.

## Figures and Tables

**Table 1:** The yield of “Eshu10” under different shading treatments.

Variety	Treatment	Yield (kg)
Eshu10	CK	1.35 ± 0.29 a
	L30	0.62 ± 0.10 b
	L50	0.52 ± 0.08 b
	L70	0.12 ± 0.02 c

Lowercase letters indicate a significant difference (p ≤ 0.05).

## Data Availability

Data are contained within the article and Supplementary Materials.

## Supplementary Materials

The following supporting information can be downloaded at: https://www.mdpi.com/article/10.3390/genes14122112/s1, Figure S1: Results of GO enrichment analyses of three up-trend clusters, top 25 items were displayed; Figure S2: Results of KEGG enrichment analyses of three up-trend clusters; Figure S3: Results of KEGG enrichment analyses of comparison-specific DEGs; Table S1: Primer sequence (5′-3′); Table S2: Summary of “Eshu10” transcriptome data; Table S3: 185, 904, and 2578 DEGs identified in three comparison groups (CKvs.L30, CKvs.L50, and CKvs.L70), respectively; Table S4: DEGs enriched in Photosynthesis-related GO terms; Table S5: DEGs enriched in antioxidant-related GO terms; Table S6: DEGs enriched in phenol and flavonoid-related pathways.
