# Supplementary figures and images for: Physiological, Photosynthetic, and Transcriptomics Insights into the Influence of Shading on Leafy Sweet Potato
Source: Genes (Basel). 2023 Nov 22;14(12):2112. doi: 10.3390/genes14122112 (PMC10742944; doi:10.3390/genes14122112)

A

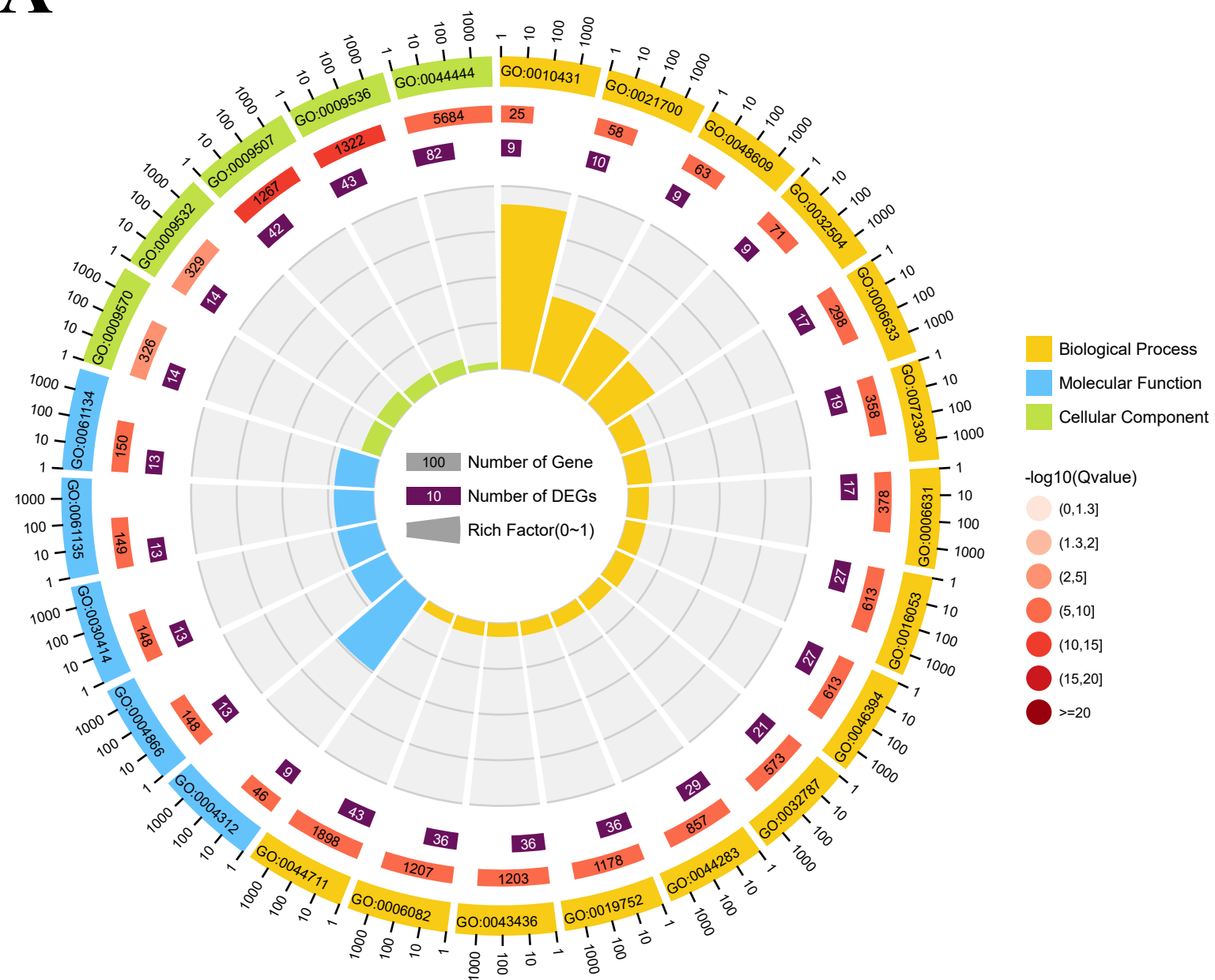

B

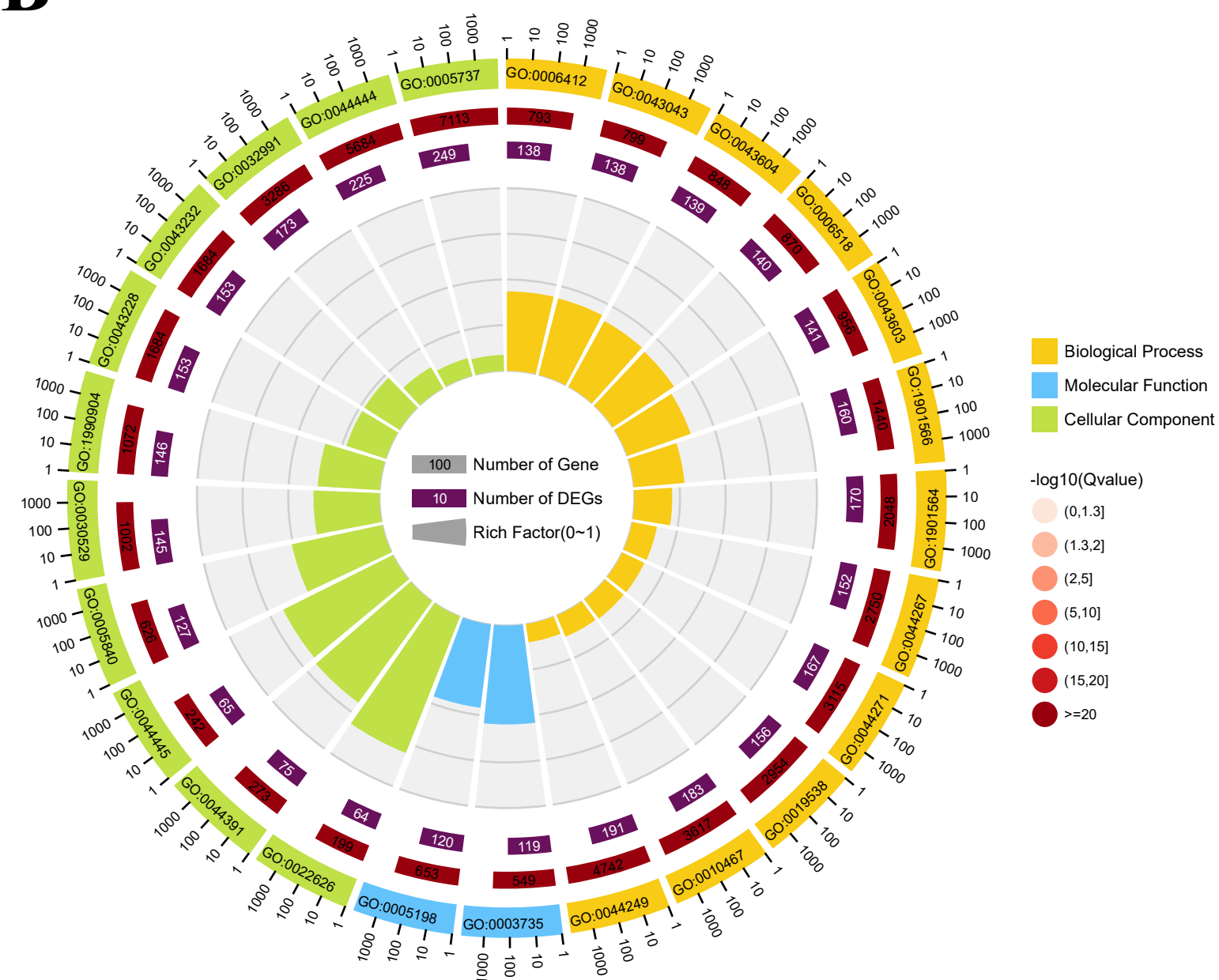

C

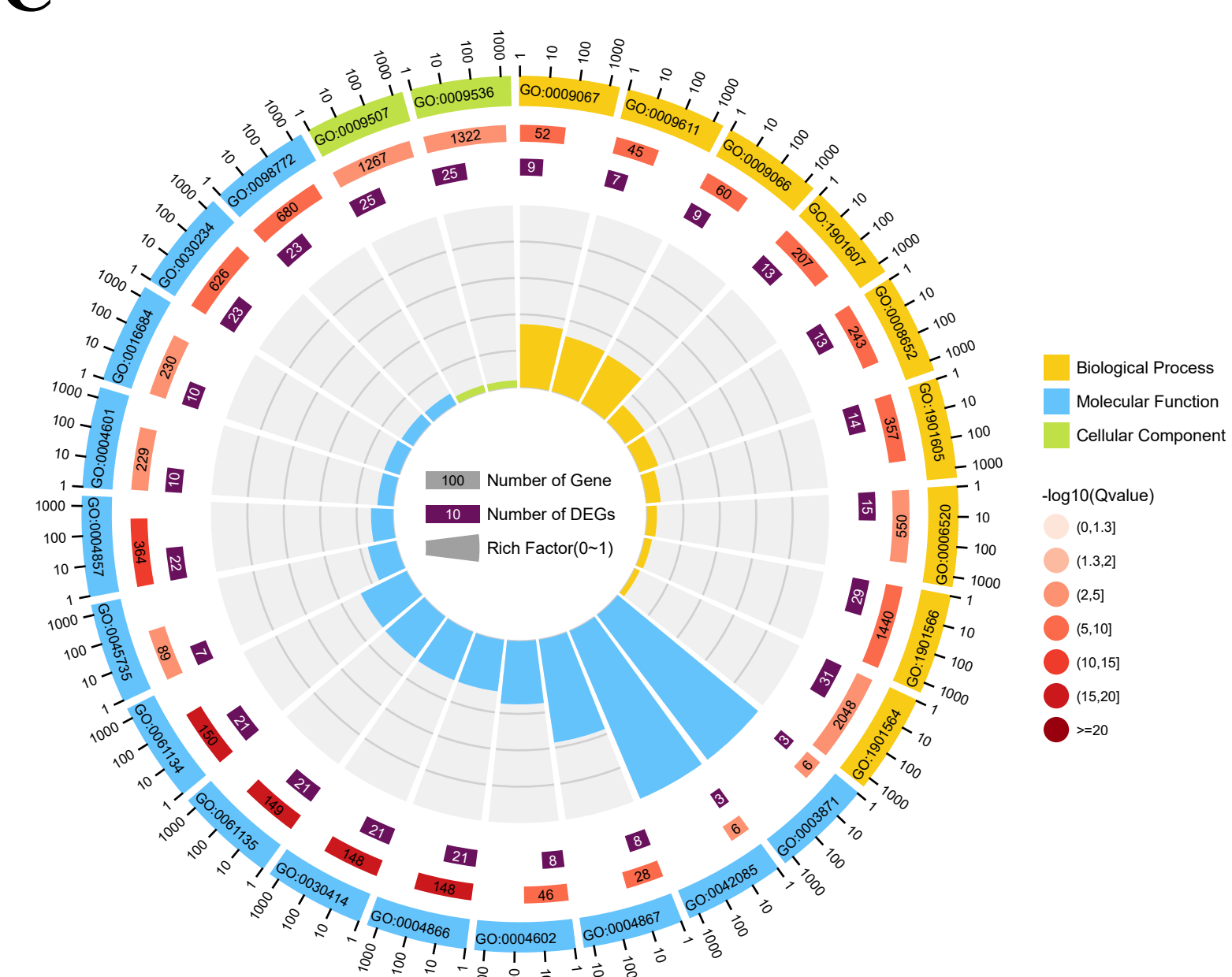

Supplement: Supplementary file 1 [file genes-14-02112-s001.zip › Supplementary Figure S1.pdf]

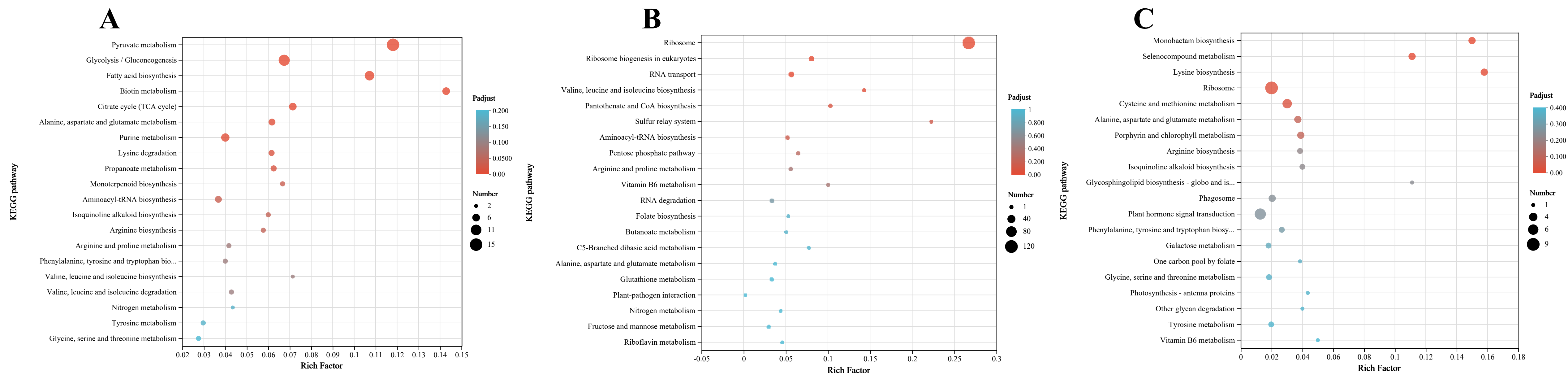

Supplement: Supplementary file 1 [file genes-14-02112-s001.zip › Supplementary Figure S2.pdf]

A

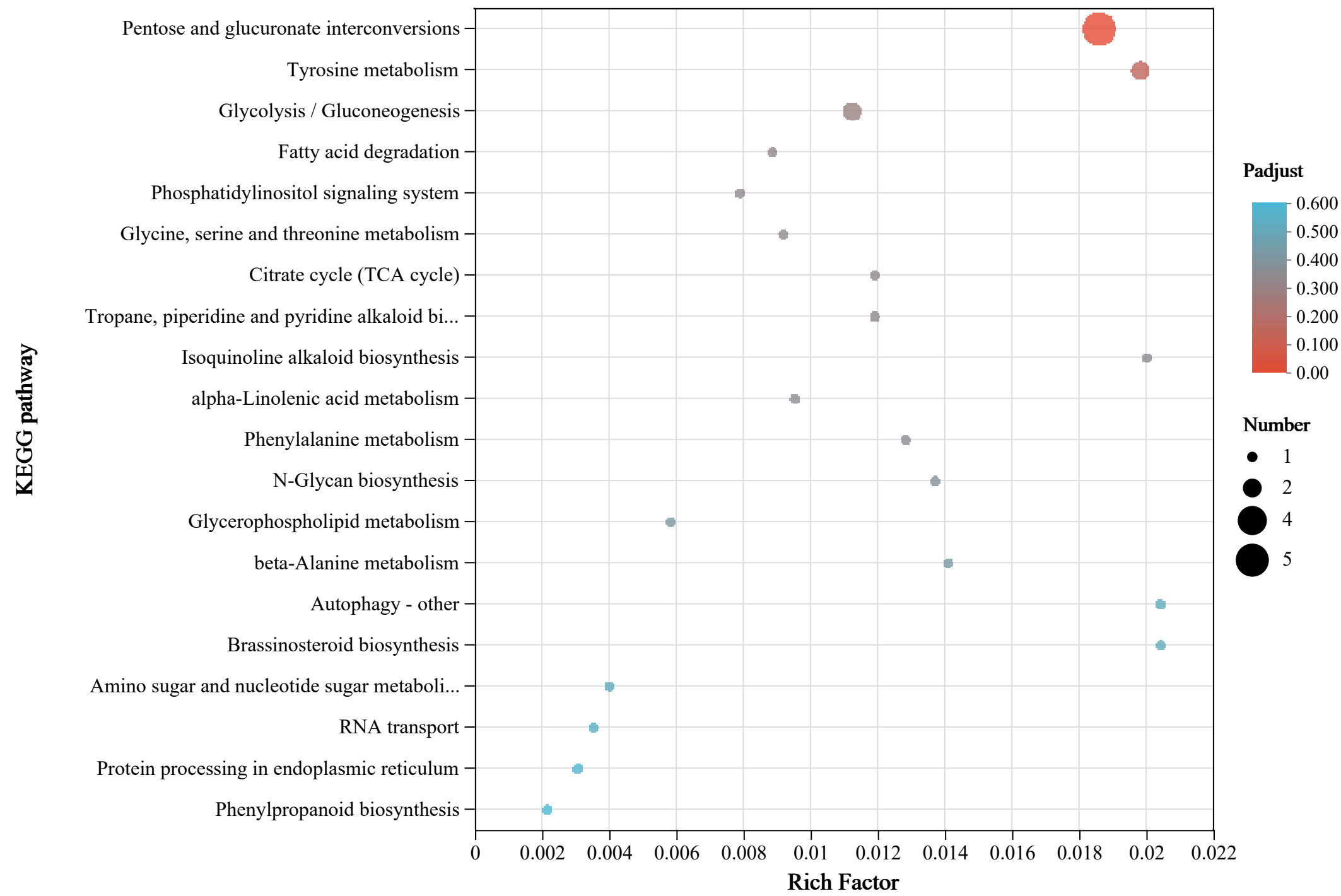

B

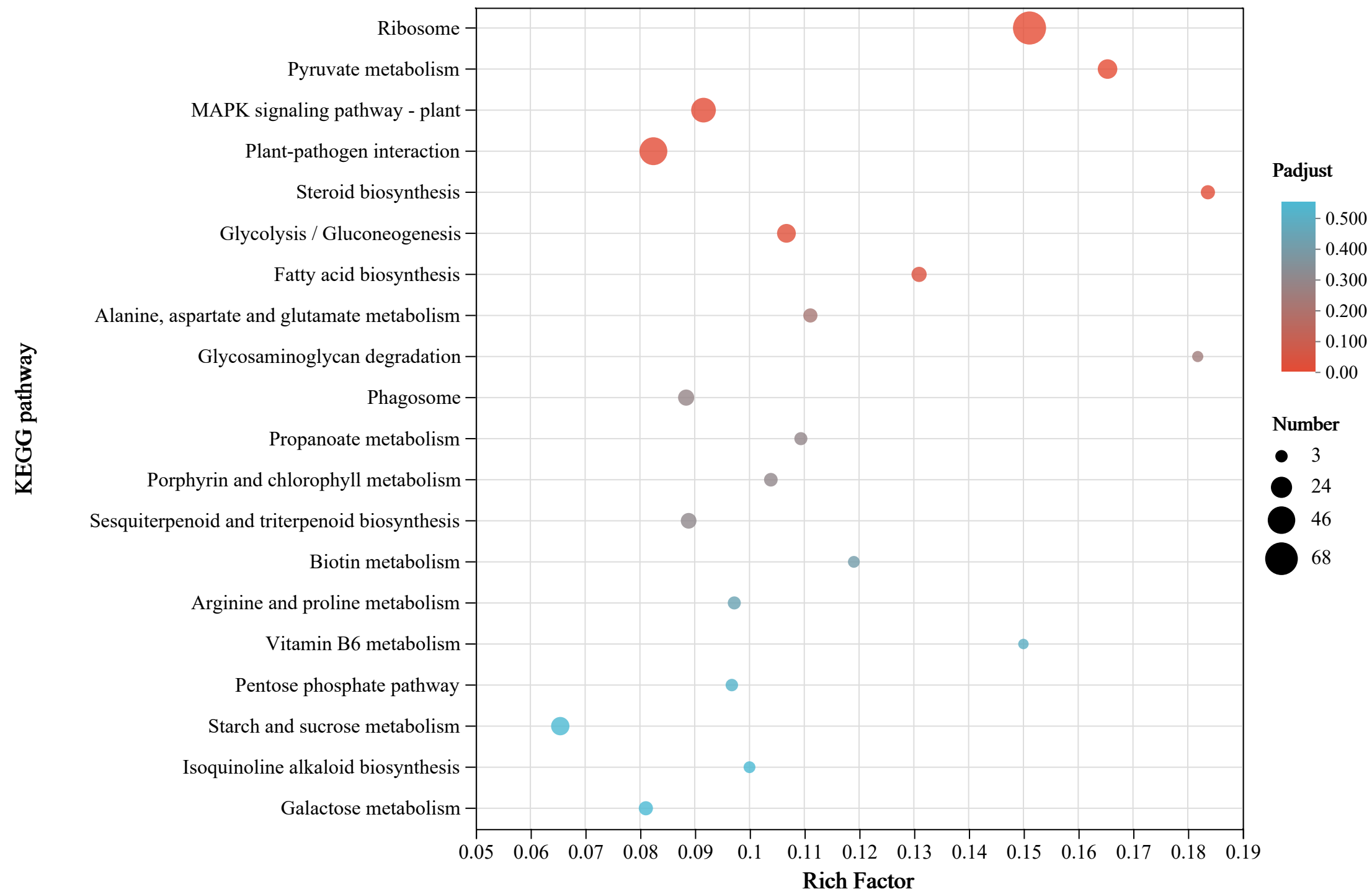

Supplement: Supplementary file 1 [file genes-14-02112-s001.zip › Supplementary Figure S3.pdf]
